# Raw Meat Consumption and Food Safety Challenges: A Survey of Knowledge, Attitudes, and Practices of Consumers in Lebanon

**DOI:** 10.3390/foods13010118

**Published:** 2023-12-29

**Authors:** Rouba Ballout, Imad Toufeili, Samer A. Kharroubi, Issmat I. Kassem

**Affiliations:** 1 Department of Nutrition and Food Sciences, Faculty of Agricultural and Food Sciences, American University of Beirut, P.O. Box 11-0236, Riad El Solh, Beirut 1107-2020, Lebanon; rb166@aub.edu.lb (R.B.); toufeili@aub.edu.lb (I.T.); 2 Center for Food Safety, College of Agricultural and Environmental Sciences, University of Georgia, Athens, GA 30602, USA; issmat.kassem@uga.edu

**Keywords:** food safety, foodborne diseases, ready-to-eat, raw meat, knowledge, attitudes, practices, Lebanon

## Abstract

A key contributor to foodborne illnesses is consuming contaminated ready-to-eat foods, including raw meats. The latter is a common practice in Lebanon, a country that suffers from widespread pollution and food safety challenges. However, studies on the safety of raw meat consumption in Lebanon are limited. In this study, an attempt was made to investigate the knowledge, attitudes, and practices (KAPs) of the Lebanese population toward the potential risk associated with the consumption of raw meats, and to identify factors that affect KAP levels. An online survey (*n* = 577) was administered to Lebanese adults aged 18 years and above to assess their KAPs. The results showed that 74.5% of the participants consumed raw meat, 44% had good food safety knowledge, and 30.7% exhibited good practices. However, more than half of the participants (61.9%) showed a positive attitude toward food safety. There was a significant association between knowledge and attitude (*p* < 0.001), attitude and practices (*p* < 0.001), and knowledge and practices (*p* < 0.001), thereby indicating that an increase in food safety education could translate into better practices in this population. Accordingly, efforts to enhance education on food safety are warranted to reduce the potential risk of food poisoning associated with raw meat consumption in Lebanon.

## 1. Introduction

Foodborne illnesses exert enormous public health and economic burdens, especially in developing countries [1]. According to the World Health Organization, an estimated 600 million people are affected and 420,000 deaths occur annually due to foodborne diseases [2]. The full burden of foodborne diseases is not well known in Lebanon, a Mediterranean country faced with serious challenges in food safety and pollution [1,3,4]. However, Lebanon is part of the MENA (Middle East and North Africa) region, which has one of the highest burdens of foodborne diseases when considering the size of the population [5]. Furthermore, food safety concerns in Lebanon have received a lot of national media attention, with outbreaks and food-associated gastrointestinal illnesses occurring regularly [1,6].

Data on food safety in Lebanon are limited due to the absence of robust surveillance systems and the lack of resources for laboratory investigations. However, it is known that a major contributor to foodborne illnesses is the high consumption of raw food, which is commonly referred to as a “risky eating habit” [7]. In Lebanon, raw red meat is an ingredient in several prized and famous dishes (such as Kebeh and Kafta) that characterize Lebanese cuisine and are extensively served in restaurants in the country and abroad. In 2013, Lebanon’s beef meat consumption was estimated at 39.63 kg per capita [8]. Despite having a high nutritional value as a source of high-quality proteins, healthy fatty acids, iron, minerals, and vitamins [9], raw red meat can harbor a variety of foodborne pathogens that can cause debilitating and life-threatening infections. For example, a study showed that the major bacterial pathogens found in meat in Pakistan include *Listeria monocytogenes*, *Escherichia coli*, *Salmonella enteritidis*, and *Shigella* sp. [10]. Furthermore, viruses such as Hepatitis A, Hepatitis E, and norovirus can cross-contaminate meat under poor hygienic conditions [11]. Additionally, parasites such as *Taenia saginata*, *Trichinella* spp, and *Toxoplasma gondii* can also be transmitted by meat to consumers and pose serious hazards, particularly in low- and middle-income countries [12]. It should be noted that meat can also be chemically contaminated with a variety of residues, including polychlorinated dibenzo-p-dioxins and dibenzofurans, polychlorinated biphenyls, polybrominated diphenyl ethers, perfluorooctane sulfonate, pesticides, metals, and veterinary drugs [13].

A recent study on the microbial contamination of red meat in Lebanon indicated that 76% and 98% of raw minced beef samples were microbiologically unsafe for consumption due to the high loads of *Escherichia coli* and fecal coliforms [6]. The workers further noted that 35% of the isolated *E. coli* were found to be multidrug-resistant (MDR) and exhibiting resistance to clinically and agriculturally important antibiotics. Further, the first nationwide analysis of food safety and acceptability data in Lebanon showed that *Staphylococcus aureus*, sulfate-reducing bacteria, *Escherichia coli*, *Listeria monocytogenes*, and *Salmonella* were frequently found on contaminated red meat samples collected from the Lebanese markets [1]. Furthermore, the consumption of raw or undercooked beef was a leading cause of campylobacteriosis in Lebanon [14]. Taken together, the data highlight the need for understanding the risks associated with the consumption of raw red meat and how to mitigate them in Lebanon. Of note, anecdotal evidence strongly suggests that the Lebanese consumer might not be well aware of these risks and their associated health impacts, which are largely responsible for the lack of reporting foodborne illnesses in the country and the ineffective food safety systems in Lebanon. Those risks could potentially be effectively mitigated by advocating the cooking of meat to eliminate certain microbiological hazards and to avoid the consumption of raw red meat, especially in vulnerable and infection-susceptible populations.

Many studies on food safety knowledge, attitudes, and practices (KAPs) among consumers and food handlers have been conducted worldwide. However, few studies address these parameters in the MENA region, and similar studies are even more scarce in Lebanon. The need for these studies is obvious, particularly in a country that suffers from serious food safety challenges, because analysis of KAP levels can identify gaps and allow for informed interventions that might reduce the burden of disease and save lives [15]. For example, only 32.7% of 994 participants in Egypt knew the increased risk of food poisoning from the consumption of raw or semi-cooked meat [16]. Also, a study of 1172 participants reported poor food safety practices (44.8%) and knowledge (53.6%) among university students in Lebanon [17]. Still, the study noted that Lebanese students outperformed their counterparts in Jordan, another MENA country, in the overall food-handling score. Indeed, the importance of risk perception in food handling, specifically perceived susceptibility and severity, was highlighted as an important driver for implementing sound food-handling behaviors by consumers [18]. To this end, warning messages on the risk of salmonellosis associated with the consumption of raw meat were shown to be particularly effective, as evidenced by the higher consumption of cooked meat by subjects who received the messages [19]. It should be noted that a positive attitude does not always translate into good practices [15,20,21], thereby suggesting the need for a follow-up with outreach and suitable resources to educate consumers on food safety risks. The aforementioned observations underscore the need for studies that rigorously address food safety knowledge and practices, especially in at-risk populations.

Evidence suggests that food safety in Lebanon is deteriorating, especially after the economic meltdown in 2019, which resulted in widespread poverty, severe power outages, and an erratic supply of clean water to sizable segments of the Lebanese population [1,13]. Preventive measures are almost absent due to a combination of inadequate infrastructure and lack of resources [1,13]. Shortages in the power supply and loss of purchasing power are also affecting the quality and safety of the food, especially meat. To our knowledge, this is the first study in Lebanon on the KAPs associated with raw red meat consumption.

The objective of the present work was to assess consumers’ awareness of the risks associated with the consumption of raw or undercooked meat, and how their practices might aggravate those risks. To this end, we assessed (i) the knowledge, attitudes, and practices of the Lebanese population toward the risk associated with the consumption of raw meat, (ii) factors associated with KAP levels and the inter-relations between the different levels, and (iii) measures to raise awareness of the risks associated with raw meat consumption and provide recommendations that could be beneficial for reducing foodborne diseases in the country.

## 2. Materials and Methods

### 2.1. Study Design and Sampling

This study was conducted between January and April 2022. It involved participants from across Lebanon. Sample size calculations showed that a minimum of 384 respondents were needed to estimate a prevalence of 50%, with a 95% CI, a margin of error of 5%, and a design effect of 1.5. To account for a 20% refusal rate, 577 respondents were included in the study. The sample size was calculated using the World Health Organization (WHO) sample size calculator [22].

### 2.2. Data Collection

An online invitation was shared and posted via different social media platforms (WhatsApp groups, Facebook pages, Instagram, and Twitter), where participants were invited to fill in a blinded online questionnaire and a consent form (Supplementary Material File S1). Once they agreed to take part in the study, participants proceeded to answer the survey (Supplementary Material File S2). The completion of the survey took approximately 10 min.

Participation in the survey was completely voluntary and anonymous. Moreover, participants were encouraged to ask questions related to the study or request further clarification before agreeing to participate in the study. Furthermore, the study was approved by the Institutional Review Board (IRB) at the American University of Beirut, and the research team was Collaborative Institutional Training Initiative (CITI)-certified.

### 2.3. Survey Format

The survey intended to evaluate knowledge, attitudes, and practices toward the safety of the consumption of raw meat of beef, lamb, and goat. The questionnaire was developed based on previous similar studies [21,23,24]. Specifically, the survey was divided into four sections. The first section included questions on the participants’ socio-demographic characteristics such as age, gender, area of residency, educational level, and total income. The second section comprised questions on the participant’s knowledge about the risks associated with the consumption of raw meat. The third section focused on the participants’ attitudes toward safe meat handling and its associated risks. The last section included questions on consumer practices that might increase the risk of contamination. The questionnaire was pilot-tested on 20 consumers to check for clarity and cultural sensitivity. Data collected during the pilot testing phase were not included in this study. A copy of the questionnaire used in data collection is provided in Supplementary Material File S2.

### 2.4. Statistical Analysis

The responses were rigorously checked for completeness, and only complete responses were included for data analysis. Out of the 720 subjects sent the questionnaire, 640 agreed to participate in this study, resulting in an 89% response rate. Of these, data were missing for 63 respondents, leaving 577 responses for analysis. Data were analyzed using the Statistical Package for the Social Sciences (SPSS) version 26.0 (SPSS Inc., Chicago, IL, USA). Descriptive statistics included means and standard deviations (SD) for continuous variables and frequencies and proportions for categorical variables. Each multiple-choice question had 1 correct answer that received a score of 1 point, while incorrect answers were assigned a score of 0 points. Total KAP scores were calculated for each participant by adding the number of correct answers. The total score was dichotomized as having either a lower or higher level of food safety KAP. For each variable, 70% was considered as the cutoff point [24]. Specifically, participants with KAP scores below 70% were considered to have low KAP levels, whereas those with scores ≥ 70% were considered to have high KAP levels. More specifically, a scale ranging between 0 and 26 (representing the total number of questions on food safety knowledge) was used to evaluate the overall knowledge of respondents. Food-handlers that obtained a total score ≤ 17 points were considered to have “insufficient” knowledge and those that had scores ≥ 18 points (≥70% accuracy) were considered to have “good” knowledge of food safety. Chi-square was used to calculate the association between two categorical variables. Simple and multiple logistic regression were applied to determine which factors were associated with the KAP levels. The sociodemographic characteristics were the independent variables, while total knowledge, attitude, and practice scores represented the dependent variables. Characteristics that showed statistical significance in the simple analysis were included in the multiple regression models as independent variables. The multiple logistic regression was used to explore the joint influence of multiple predictors on the KAP levels whilst controlling for potentially confounding variables that have been used in the model. Results from the logistic regression analyses were expressed as odds ratios (OR) with 95% CI. For all analyses, a *p*-value of less than 0.05 was considered statistically significant.

## 3. Results

### 3.1. Sociodemographic Characteristics of Participants

The sociodemographic characteristics of the study population are presented in Table 1. Results showed that more than half of the participants were women (69.7%) and almost half of the participants were aged between 18 and 29 (48.5%). The study sample included consumers from all over Lebanon, mostly from the capital, Beirut (42.3%), and Mount Lebanon (33.4%). More than one-third of the participants held a bachelor’s degree (37.4%) and 40.4% had a master’s degree. Around 41% of the participants had a monthly income of more than 5,000,000 Lebanese Lira (L.L.) (225$). Furthermore, more than half of the respondents or someone they know had experienced symptoms of foodborne disease in the past 6 months (64.3%). More than two-thirds (67.1%) of the participants self-reported having a good level of food safety knowledge, while 22% considered their knowledge to be excellent and 10.9% weak. Most of the consumers obtained their food safety information from the internet and social media (52.5%).

Almost 15% of the participants consumed raw meat at least once per week, nearly half of the participants did not consume it weekly (48.2%), while 25.5% did not consume raw meat at all. The Lebanese consumer’s favorite raw meat dish was kibbeh (39%). They also preferred to buy meat from their neighborhoods (62.6%), mostly from butcher shops (83.2%).

### 3.2. Food Safety Knowledge

Table 2 summarizes the food (raw meat) safety knowledge of Lebanese consumers. Overall, knowledge was unsatisfactory, because the mean score was 16.99 ± 5.231, which is below the cutoff of 18.2 (≥70% accuracy). The majority of the consumers knew what food poisoning is (92.4%), and that they can get seriously sick and potentially die from eating contaminated food (91.2%). Furthermore, the majority knew that food could get contaminated at home and/or the market (87%) and that washing hands can reduce the risk of contamination during food preparation (87%). More than two-thirds (68.5%) of the respondents knew which population was more susceptible to food poisoning. Almost half (53.7%) of the study population were aware that the consumption of raw meat could pose serious economic losses. More than half of the participants (51%) knew about cross-contamination. Most of the participants knew that proper cooking of the meat was the only way to ensure that they would not get poisoned (76.9%). Additionally, 52% and 76.6% knew the optimal time and temperature for storing raw meat in the fridge, respectively, and 64% knew that raw meat dishes that look and smell fresh can also be contaminated and make them sick. Also, 67.8% knew that raw meat cannot be kept at room temperature for more than 2 h. Most of the participants (70.5%) knew that they should check the temperature of the meat during long power cuts to decide whether to keep it or discard it. However, only 43.5% of them knew that freezing can prevent the growth of pathogens on raw meat. Although a total of 73.8% knew the risks associated with pathogens that can occur on raw meat, lower percentages were noted when asking about those foodborne pathogens. Almost half of the participants were not familiar with foodborne parasites, viruses (e.g., Hepatitis A virus), or the bacterium Helicobacter pylori that can be found on raw meat (55.6, 57.7, and 53.7%, respectively). In contrast, almost half of them had heard of E. coli O157: H7 and the disease caused by Campylobacter (56 and 50.8%, respectively). Salmonella was the most popular among the consumers (78.2%). More than half of the respondents (56%) knew that there is a food safety law in Lebanon, but it is not implemented.

### 3.3. Food Safety Attitude

Table 3 summarizes the food safety attitudes of Lebanese consumers. The overall attitude of the consumers was acceptable, with a total mean score of 9.75 ± 2.608, which is close to the cutoff of 9.8 (≥70%accuracy). The majority of the respondents had a positive attitude when it comes to the use of gloves (82.8%) and the separation of raw meat from other types of food (84.2%). The majority of the participants (86.5% and 89.4%) agreed, respectively, that preventing contamination is one of their responsibilities and that their purchasing habits could affect food safety. A high percentage (76.3%) agreed that the government, markets, and consumers are responsible for keeping the food safe. The majority also agreed that visual inspection is not enough to determine if the food is safe (80.6%), and that high-risk populations should not consume raw meat (85.4%). More than half (59.1%) of the consumers were aware that mincing meat at home could have a lower risk of contamination in comparison to buying already chopped and minced raw meat. However, almost half of the participants indicated that washing raw meat was an essential step (47.5%). In addition, 58.9% of the participants had a negative attitude related to the addition of raw vegetables and spices to raw meat, claiming that this would not lead to the cross-contamination of the meat. Additionally, 66.9% did not object to the use of grinding machines when buying ground raw meat at the store or butcher shop. Finally, 74.2% of the respondents did not have confidence in the safety of raw meat in Lebanon, and 54.9% were inclined to stop eating raw meat after becoming aware of the risks associated with this consumption.

### 3.4. Food Safety Practices

The total mean score of 7.09 ± 2.551, being lower than the cut-point of 8.4, reflected unsatisfactory food safety practices (Table 4). However, good practices such as washing hands (93.9%), not handling raw meat while sick (80.1%), and safely defrosting raw meat (67.5%) were prevalent. Only a few respondents favored meat with lower prices after the economic meltdown in the country (23.6%). The majority of the respondents would not offer raw meat to the children and elderly in their household (70.5%). Almost half of the respondents (51.1%) bought raw meat at the end of their supermarket shopping and sought food safety certificates in the outlet where they purchased meat (52%). In comparison, only 8.5% of the respondents use a thermometer while cooking meat, and a few respondents wore gloves while preparing raw meat dishes (37.4%). The majority did not store raw meat on the appropriate shelf in the refrigerator (75.9%). Additionally, only 48% applied appropriate cleaning to their kitchen countertops, and 45.1% checked their refrigerator temperature.

### 3.5. Simple and Multiple Logistic Regression Analyses

Simple logistic regression showed that four predictors were significantly associated with the participants’ knowledge scores (Table 5), including 1—the area of residency (OR = 2.288, *p* = 0.048), where participants from North Lebanon were more likely to have better knowledge than participants residing in Beirut, 2—education level (PhD: OR = 6, *p* = 0.019; Master’s Degree: OR = 5.288, *p* = 0.012), 3—the way participants rated their food safety knowledge (Good: OR = 0.282, *p* = 0.000; Weak: OR = 0.09, *p* = 0.000), and 4—the source of food safety information (Family and Friends: OR = 0.561 *p* = 0.033; University: OR = 5.427, *p* = 0.000; No source: OR = 0.3, *p* = 0.003). Multiple regression analyses also showed that the way participants rated their food safety knowledge (Good: OR = 0.372, *p* < 0.001; Weak: OR = 0.164, *p* < 0.001), and the source of food safety information (University: OR = 3.495, *p* < 0.001; No source: OR = 0.407, *p* = 0.037) were significantly associated with their knowledge score.

Simple logistic regression analysis also showed that the variables significantly associated with the likelihood of having a positive attitude in the study population were similar to those associated with knowledge level, including the area of residency (OR = 1.492, *p* = 0.047), education level (OR = 3.018, *p* = 0.040), the way participants rated their food safety knowledge (Good: OR = 0.458, *p* = 0.001; Weak: OR = 0.264, *p* = 0.000), and the source of food safety information (Family and Friends: OR = 0.602, *p* = 0.041; University: OR = 3.200, *p* = 0.000; No source: OR = 0.479, *p* = 0.021). The results of the multiple logistic analysis showed that the participants who reported having a weak level of knowledge were less likely to have a positive attitude compared to those who reported having excellent knowledge (OR = 0.476, *p* = 0.039 (Table 5). Additionally, participants who acquired their food safety information from a university were more likely to have a positive attitude towards food safety compared to those who got it from the internet and social media (OR = 2.355, *p* = 0.004).

Unlike the other variables, practices were shown to be associated with the monthly income of the participants (OR = 2.262, *p* = 0.019) (Table 5). However, good practices were associated with the participants’ educational level (Other: OR = 12.923, *p* = 0.042; Master’s Degree OR = 8.342, *p* = 0.021), the way they rated their food safety knowledge (Good: OR = 0.332, *p* = 0.000; Weak: OR = 0.224, *p* = 0.000), and the source of food safety information (University: OR = 2.083, *p* = 0.001; No source: OR = 0.428, *p* = 0.048). Multiple regression analysis revealed a significant difference in only the participant’s rating of their food safety knowledge. Good and weak ratings were, respectively, 0.409 (OR = 0.409, *p* = 0.000) and 0.341 (OR = 0.341, *p* = 0.007) less likely to have good safety practices compared to an excellent rating.

### 3.6. Association between the Different KAP Scores

Table 6 shows a significant association between the different combinations of KAP levels. The odds of having a positive attitude given good knowledge were (OR = 5.492) higher compared to when participants have insufficient knowledge. Furthermore, good practices were more likely when participants had good food safety knowledge in comparison to insufficient knowledge (OR = 4.027). Similarly, good practices were more likely to be encountered when participants had positive attitudes in comparison to negative attitudes (OR = 2.577).

## 4. Discussion

Recent spikes in the incidences of food poisoning and contamination in Lebanon have been well covered in the national media and scientific reports [1]. Currently, food safety in Lebanon is challenged by several factors, including weak governance, widespread pollution, and an unfolding severe economic crisis that paralyzed many vital sectors in Lebanon [3,25]. These conditions resulted in a need to re-evaluate food preferences and habits that might pose an elevated risk, to guide the population during these challenging times. A major food safety concern is the consumption of raw meat incorporated into popular and national dishes in Lebanon. These concerns were spurred by reports that showed widespread contamination of raw beef samples in Lebanon [6]. Taken together, it was necessary to conduct this study that assessed the knowledge, attitudes, and practices of Lebanese consumers about the safety of raw meat.

The present findings indicate that the participants had an overall unsatisfactory level of knowledge and good practices related to the safety of raw meat consumption. The observed unsatisfactory food safety knowledge among consumers is shared by other countries in the Middle East and North Africa (MENA) region, including Iran and Jordan [21,26], thereby highlighting the need for food safety training and awareness initiatives. An insufficient level of knowledge was also corroborated in a previous study that targeted Lebanese university students [17]. However, it should be noted that the present work involved a more diverse and larger number of participants and included, in many instances, questions about etiologic agents of foodborne disease in comparison to other studies in the region [27]. The latter might have also resulted in a lower number of satisfactory responses, especially about food safety knowledge [28]. Indeed, the majority of the participants in our study had little knowledge of the pathogens that can be transmitted through raw meat, which corroborated observations from Iran [21]. However, it should be noted *Salmonella* was found to be well known among Lebanese consumers (*n* = 451, 78.2%), likely due to media focusing on *Salmonella*, which is a leading agent of foodborne infections worldwide. The total mean of food safety attitude was 9.75 ± 2.608, thus reflecting a positive attitude among consumers. This finding has also been reported in studies from Ethiopia [24], Malaysia [29], and Iran [21]. Fortunately, attitude is one of the key elements that affect food safety and the practices that help reduce the risk of foodborne diseases [30]. It is perhaps the innate need to require food to be safe and nutritious that drives the positive attitude toward food safety across different countries. Therefore, it was not surprising that the participants were concerned about the safety of raw meat in Lebanon and that 317 (54.9%) would refrain from eating raw meat if they were aware of the risks associated with it.

There was an unsatisfactory level of good practices associated with raw meat safety, with a total mean of correct answers of 7.09 ± 2.551 (<70%) (Table 4). For example, few participants (*n* = 49, 8.5%) used a thermometer while cooking meat, similar to observations reported from Malaysia [29]. Furthermore, the majority of participants did not store raw meat on the lower shelf of their refrigerator, thus increasing the risk of cross-contamination with other foods (*n* = 438, 75.9%). While this poses a concern, it is noteworthy that bad practices can be remediated by outreach and educational programs [28]. In this connection, it should be noted that assessing food safety practices would have been more informative through an observational study to avoid any bias in participants’ answers.

The data also showed a significant association between the level of knowledge and the source of food safety information and the way the participants rated their food safety knowledge. Participants with graduate degrees had a better level of knowledge than those who obtained their food safety information from the internet and social media, presumably due to the questionable quality and accuracy of some information disseminated via these outlets. Similarly, participants who rated their food safety knowledge as excellent were more likely to have a positive attitude. Again, only the source of information affected the level of good practice (*p* < 0.05), further confirming that inaccurate sources lead to wrong practices and an increase in the risk of food poisoning. Unlike other studies, where knowledge increased with age and was related to the participants’ gender, no association between knowledge and age was found in the present study [16,31].

In this study, a significant association was found between the different KAP elements, which was different from observations in Malaysia, where only attitude toward food safety had a direct effect on practices [29]. This finding suggests that better food safety knowledge and understanding of risks associated with the consumption of raw meat might be translated to better practices in the study population. This in turn should allow for potentially successful interventions via outreach and education programs. For example, preventive messages on the risk of salmonellosis associated with the consumption of raw meat were effective, as evidenced by the increase in the consumption of cooked meat, instead of raw meat, by individuals who received the message [19]. Despite their good food safety knowledge, 45.1% of the participants indicated that they were not ready to stop eating raw meat, thus highlighting the deeply rooted preference and cultural inclination for consuming national dishes incorporating raw meat. The aforementioned drivers underpinning the consumption of raw-meat-incorporating dishes indicate that interventions must go beyond simple messaging and be more rigorous, to include well-thought-out and easy-to-implement programs that assure the safety of raw meat to curb the grave consequences associated with the high-risk practice of raw meat consumption. This knowledge–practice gap was highlighted in a previous study that showcased the limitations of the KAP model in food safety: precisely, the presence of well-known cognitive factors that affect food safety practices and are not accounted for in the model, such as beliefs and habits [32].

Several limitations might have affected our observations. For example, online surveys could lead to selection-bias, because not all groups in a population might have equal access to the internet or participate in social media platforms. The latter might include rural communities, disenfranchised groups, and/or the elderly population, which are all prone to foodborne infections. Despite limitations, our study can be used to justify the development of direct-to-consumer interventions such as messaging and educational material that target risky foods such as raw meat.

## 5. Conclusions

In conclusion, Lebanese consumers had gaps in KAPs related to the safety of consuming raw meat, mainly the potential of cross-contamination, proper storage of raw meat, and the identity and variety of foodborne pathogens that could be transmitted via raw meat among others. Therefore, there is a need for interventions to limit the consumption of highly risky foods in a country that suffers from multiple food safety challenges. To this end, the present work can be used to develop appropriate safety measures (educational and outreach programs) to protect consumers against potentially debilitating foodborne infections. To succeed, these efforts should engage multiple stakeholders, including the government and other public and private entities that can devise and deliver a consolidated educational program on food safety in general and raw meat safety in particular, accessible to all the population in Lebanon.

## Figures and Tables

**Table 1:** Sociodemographic characteristics of the study population (*n* = 577).

Characteristics		*n* (%)
Gender	Men	175 (30.3)
	Women	402 (69.7)
Age	18–29	280 (48.5)
	30–39	135 (23.4)
	40–49	86 (14.9)
	50–59	61 (10.6)
	60+	15 (2.6)
Area of residency	Beirut	244 (42.3)
	South	80 (13.9)
	North	27 (4.7)
	Mount Lebanon	193 (33.4)
	Bekaa	23 (4.0)
	Other	10 (1.7)
Education level	Primary school	15 (2.6)
	High school	63 (10.9)
	Bachelor degree	216 (37.4)
	Master degree	233 (40.4)
	Technical degree	25 (4.3)
	Other	25 (4.3)
Monthly income	<1,000,000 L.L	49 (8.5)
	1,000,000–3,000,000 L.L	152 (26.3)
	3,000,000–5,000,000 L.L	122 (21.1)
	≥5,000,000 L.L	237 (41.1)
	Other	17 (2.9)
Experienced symptoms of food-borne disease	Yes	371 (64.3)
	No	206 (35.7)
Rating their food safety knowledge	Excellent	127 (22.0)
	Good	387 (67.1)
	Weak	63 (10.9)
Source of food safety information	Internet/Social media	303 (52.5)
	Family/Friends	83 (14.4)
	TV	11 (1.9)
	University	113 (19.6)
	No source	47 (8.1)
	Other	20 (3.5)
How often do you eat raw meat per week?	More than once/week	39 (6.8)
	Once/week	48 (8.3)
	Not every week	278 (48.2)
	Do not eat raw meat	147 (25.5)
	Other	65 (11.3)
My favorite raw meat dish	Kibbeh ^1^	225 (39.0)
	Kafta ^2^	85 (14.7)
	Liver	57 (9.9)
	All	110 (19.1)
	Other	100 (17.3)
Do you prefer to buy raw meat for consumption local or imported?	Local	361 (62.6)
	Imported	90 (15.6)
	Both	111 (19.2)
	Other	15 (2.6)
Where do you prefer to buy your raw meat?	Butcher shops	480 (83.2)
	Markets	91 (15.8)
	Other	6 (1.0)

^1^ A national dish in Lebanon. It is usually prepared by pounding wheat bulgur with the meat and served with different garnishes such as fresh mint leaves, green onions, and olive oil. ^2^ Raw meat minced with fresh parsley, basil, spring onions, and spices.

**Table 2:** Key indicators of the participants’ food safety knowledge.

Question	Right Answer *n* (%)	Wrong Answer *n* (%)
Do you know that people can get seriously sick and die from eating contaminated food?	526 (91.2)	51 (8.8)
Do you know what food poisoning is?	533 (92.4)	44 (7.6)
Do you know which population is more susceptible to food poisoning?	395 (68.5)	182 (31.5)
In your opinion can food get contaminated at:	502 (87)	75 (13)
The consumption of raw meat could pose serious economic losses to consumers	310 (53.7)	267 (35.4)
Form of cross-contamination that could happen due to bad practices or bad storage conditions	294 (51)	283 (49)
Can a raw meat dish that looks and smells fresh make you sick?	373 (64.6)	204 (35.4)
Does freezing (<−18 °C) prevent the growth of pathogens on raw meat?	251 (43.5)	326 (56.5)
The only way to ensure that you won’t get food poisoning due to raw meat is to:	444 (76.9)	133 (23.1)
What is the optimal temperature for storing raw meat?	442 (76.6)	135 (23.4)
Regular washing of hands reduces the risk of contamination	502 (87)	75 (13)
Raw meat can be kept at room temperature for:	391 (67.8)	186 (32.2)
During a long electricity cut-off, what should you do with your stored raw meat?	407 (70.5)	170 (29.5)
How long can you store raw meat in the fridge before consuming it?	300 (52)	277 (48)
Do you know that some pathogens in raw meat can lead to kidney failure, sepsis, bloody diarrhea, and even death?	426 (73.8)	151 (26.2)
Parasites that could be found in raw meat dishes	256 (44.4)	321 (55.6)
Hepatitis A virus that can cause liver inflammation can be found in raw meat dishes	244 (42.3)	33 (57.7)
*Helicobacter pylori* a bacterium that can cause peptic ulcer disease and gastritis can be transmitted through raw meat dishes	267 (46.3)	310 (53.7)
*Salmonella* bacteria that infect the intestinal tract can be found in raw meat dishes	451 (78.2)	126 (21.8)
*E. coli* O157: H7 can cause serious illness in humans by producing toxins that can severely damage the lining of your intestines and kidneys. Can you get this bacterium from eating raw meat dishes?	323 (56)	254 (44)
Campylobacteriosis (the most common cause of bacterial diarrhea and can cause stunting in children) could be transmitted through raw meat dishes	293 (50.8)	284 (49.2)
Do you have any information on the consequences of antibiotic-resistant bacteria that could be transmitted through food/raw meat?	287 (49.7)	290 (50.3)
Can contamination happen in an unclean grounding machine?	501 (86.8)	76 (13.2)
Do you know that traditional ways of preparing raw meat dishes are more dangerous than modern ways?	346 (60)	231 (40)
Do you know that the longer the preparation time the more it is likely to contaminate the meat?	414 (71.8)	163 (28.2)
Are there any food safety law/efforts in Lebanon that protects you as a consumer?	323 (56)	254 (44)
Total mean of correct answer: 16.99 ± 5.231		

**Table 3:** Key indicators of the participants’ attitude.

Question	Positive Attitude *n* (%)	Negative Attitude *n* (%)
Using gloves is important in reducing the risk of food contamination	478 (82.8)	99 (17.2)
Preventing contamination is your responsibility	499 (86.5)	78 (13.5)
Visual inspection is enough to determine if the food is safe	465 (80.6)	112 (19.4)
Separate raw meat from ready-to-eat food to prevent cross-contamination	486 (84.2)	91 (15.8)
Your purchasing habits affect food safety	516 (89.4)	61 (10.6)
People with a high risk of contamination should not consume raw meat	493 (85.4)	84 (14.6)
Washing raw meat is an essential step	303 (52.5)	274 (47.5)
Adding vegetables/spices to your raw meat at home could lead to cross-contamination	237 (41.1)	340 (58.9)
Bacteria found in raw meat are not harmful	433 (75)	144 (25)
Mincing the meat at home could have a lower risk of contamination than buying already chopped and minced raw meat	341 (59.1)	236 (40.9)
Currently, how confident are you in the safety of raw meat in Lebanon?	428 (74.2)	149 (25.8)
Who do you think is responsible for keeping the food safe?	440 (76.3)	137 (23.7)
When you buy ground raw meat at the store or butcher shop, do you mind if they use grounding machines?	191 (33.1)	386 (66.9)
If you are aware of the risks associated with the consumption of raw meat, would you refrain from eating it?	317 (54.9)	260 (45.1)
Total mean of correct answer: 9.75 ± 2.608		

**Table 4:** Key indicators of the participants’ practices.

Question	Good Practice *n* (%)	Bad Practice *n* (%)
During your supermarket shopping, when do you buy raw meat?	295 (51.1)	282 (48.9)
Do you use a thermometer while cooking the meat?	49 (8.5)	528 (91.5)
Do you prepare and handle raw meat while you are sick?	462 (80.1)	115 (19.9)
Do you wear gloves while preparing your food?	216 (37.4)	361 (62.6)
Do you wash your hands before and after handling raw meat?	542 (93.9)	35 (6.1)
After preparing raw meat, how do you clean your kitchen counters?	277 (48)	300 (52)
Do you check the temperature of your refrigerator often during the day?	260 (45.1)	317 (54.9)
Where do you store your raw meat in the refrigerator?	139 (24.1)	438 (75.9)
After the crisis, did you aim for meat with lower prices?	441 (76.4)	136 (23.6)
How do you defrost frozen meat?	389 (67.5)	188 (32.6)
Would you offer raw meat to children and the elderly in your household?	407 (70.5)	170 (29.5)
Do you look for food safety certificates in the market or shop where you buy your meat?	300 (52)	277 (48)
Total mean of correct answer: 7.09 ± 2.551		

**Table 5:** Simple and multiple logistic regression for the association of characteristics of the study population with levels of food safety knowledge, attitudes, and practices.

	Knowledge		Attitude		Practices	
	Simple logistic regression OR, (95% CI), *p*-value	Multiple logistic regression OR, (95% CI), *p*-value	Simple logistic regression OR, (95% CI), *p*-value	Multiple logistic regression OR, (95% CI), *p*-value	Simple logistic regression OR, (95% CI), *p*-value	Multiple logistic regression OR, (95% CI), *p*-value
Gender						
Women	1		1		1	
Men	0.764 (0.532, 1.096), 0.143		0.703 (0.489, 1.009), 0.056		0.708 (0.476, 1.054), 0.089	
Age						
60+	1		1		1	
18–29	0.647 (0.228, 1.833), 0.412		0.667 (0.22, 2.001), 0.470		0.679 (0.225, 2.055), 0.494	
30–39	0.743 (0.255, 2.165), 0.586		1.609 (0.512, 5.055), 0.415		1.140 (0.368, 3.525), 0.821	
40–29	0.835 (0.278, 2.507), 0.748		0.844 (0.265, 2.689), 0.744		1.071 (0.335, 3.423), 0.907	
50–59	0.530 (0.170, 1.654), 0.274		0.484 (0.148, 1.583), 0.230		1.128 (0.342, 3.723), 0.843	
Area of residency						
Beirut	1	1	1	1	1	
South	0.808 (0.481, 1.357), 0.420	0.910 (0.506, 1.636)	1.006 (0.602, 1.680), 0.982	1.050 (0.607, 1.815), 0.862	0.774 (0.431, 1.390), 0.391	
North	**2.288 (1.007, 5.203), 0.048**	2.333 (0.923, 5.898), 0.073	1.027 (0.458, 2.307), 0.948	0.895 (0.377, 2.130), 0.803	1.462 (0.638, 3.350), 0.369	
Mount Lebanon	1.128 (0.771, 1.650), 0.534	1.021 (0.661, 1.576), 0.927	**1.492 (1.005, 2.216), 0.047**	1.354 (0.887, 2.068), 0.160	1.479 (0.989, 2.212), 0.057	
Bekaa	1.234 (0.524, 2.906), 0.630	1.244 (0.453, 3.417), 0.672	1.324 (0.541, 3.241), 0.539	1.267 (0.475, 3.383), 0.637	0.877 (0.332, 2.317), 0.792	
Other	0.897 (0.247, 3.261), 0.869	0.734 (0.160, 3.366), 0.691	0.706 (0.199, 2.504), 0.590	0.604 (0.152, 2.395), 0.473	-	
Education level						
Primary school	1	1	1	1	1	1
High school	1.250 (0.311, 5.027), 0.753	1.114 (0.256, 4.858), 0.885	0.752 (0.242, 2.335), 0.622	0.635 (0.195, 2.075), 0.453	5.174 (0.631, 42.405), 0.126	5.565 (0.655, 47.272), 0.116
Bachelor degree	2.545 (0.698, 9.288), 0.157	2.477 (0.630, 9.743), 0.194	1.570 (0.550, 4.485), 0.400	1.316 (0.440, 3.935), 0.623	4.783 (0.615, 37.215), 0.135	4.700 (0.577, 38.323), 0.148
Mater degree	**5.288 (1.437, 19.018), 0.012**	3.627 (0.918, 14.333), 0.066	**3.018 (1.051, 8.662), 0.040**	1.943 (0.641, 5.888), 0.240	**8.342 (1.078, 64.548), 0.042**	6.259 (0.765, 51.232), 0.087
Technical degree	1 (0.202, 4.995), 1	0.956 (0.176, 5.182), 0.958	1.055 (0.293, 3.803), 0.935	0.909 (0.238, 3.476), 0.889	3.500 (0.368, 33.308), 0.276	3.196 (0.316, 32.378), 0.325
Other (Ph.D.…)	**6 (1.343, 26.808), 0.019**	4.604 (0.923, 22.972), 0.063	3.619 (0.921, 14.214), 0.065	2.369 (0.569, 9.870), 0.236	**12.923 (1.468, 113.773), 0.021**	9.985 (1.061, 93.972), 0.44
Monthly income						
<1,000,000	1		1		1	1
1,000,000–3,000,000	0.693 (0.359, 1.338), 0.275		0.568 (0.181, 1.779), 0.332		1.441 (0.659, 3.148), 0.360	1.149 (0.653, 3.395), 0.344
3,000,000–5,000,000	0.990 (0.507, 1.936), 0.978		0.590 (0.208, 1.779), 0.323		1.446 (0.649, 3.223), 0.367	1.307 (0.557, 3.068), 0.538
≥5,000,000	1.368 (0.735, 2.543), 0.323		0.734 (0.255, 2.114), 0.567		**2.262 (1.076, 4.756), 0.031**	1.801 (0.810, 4.002), 0.149
Other	1.5 (0.495, 4.541), 0.473		1.443 (0.513, 4.063), 0.487		2.127 (0.632, 7.157), 0.223	1.804 (0.481, 6.757), 0.381
Knowledge rating						
Excellent	1	1	1	1	1	1
Good	**0.282 (0.183, 0.434), 0.000**	**0.372 (0.229, 0.605), 0.000**	**0.458 (0.290, 0.723), 0.001**	0.634 (0.386, 1.042), 0.072	**0.332 (0.219, 0.504), 0.000**	**0.409 (0.259, 0.645), 0.000**
Weak	**0.09 (0.043, 0.192), 0.000**	**0.164 (0.073, 0.369), 0.000**	**0.264 (0.139, 0.502), 0.000**	**0.476 (0.235, 0.964), 0.039**	**0.224 (0.224, 0.109), 0.000**	**0.341 (0.156, 0.743), 0.007**
Source of info						
Internet/Social	1	1	1	1	1	1
Family/Friends	**0.561 (0.329, 0.955), 0.033**	0.665 (0.380, 1.166), 0.155	**0.602 (0.369, 0.980), 0.041**	0.653 (0.392, 1.085), 0.100	0.776 (0.443, 1.359), 0.375	0.855 (0.478, 1.528), 0596
TV	0.836 (0.24, 2.918), 0.779	0.925 (0.248, 3.449), 0.907	0.539 (0.161, 1.806), 0.316	0.49 (0.139, 1.1734), 0.269	-	-
University	**5.427 (3.273, 8.999), 0.000**	**3.495 (2.035, 6.004), 0.000**	**3.200 (1.857, 5.514), 0.000**	**2.355 (1.324, 4.190), 0.004**	**2.083 (1.435, 3.251), 0.001**	1.329 (0.812, 2.174), 0.258
No source	**0.3 (0.136, 0.664), 0.003**	**0.407 (0.175, 0.947), 0.037**	**0.479 (0.257, 0.893), 0.021**	0.567 (0.292, 1.101), 0.094	**0.428 (0.428, 0.184), 0.048**	0.484 (0.201, 1.164), 0.105
Other	0.788 (0.306, 2.031), 0.622	0.544 (0.198, 1.493), 0.237	1.509 (0.564, 4.036), 0.412	1.031 (0.373, 2.850), 0.953	2.443 (2.443, 0.983), 0.055	1.857 (0.714, 4.832), 0.205

Estimates shown in bold are those that are statistically significant at *p* < 0.05.

**Table 6:** Association between the level of food safety KAPs.

	Total (*n* = 577)	Positive Attitude (%)	Negative Attitude (%)	Significance; OR (CI)	Good Practices (%)	Bad Practices (%)	Significance; OR (CI)
Good Knowledge	254	209 (82)	45 (18)	***p* = 0.000** Χ2 = 80.136	119 (46.9)	135 (53.1)	***p* = 0.000** Χ2 = 55.820
Insufficient Knowledge	323	148 (46)	175 (54)	5.492 (3.720, 8.107)	58 (18)	265 (82)	4.027 (1.730, 3.839)
Positive Attitude	357				135 (37.8)	222 (62.2)	***p* = 0.000** Χ2 = 22.441
Negative Attitude	220				42 (19.1)	178 (80.9)	2.577 (1.730, 3.839)

Estimates shown in bold are those that are statistically significant at *p* < 0.05.

## Data Availability

The data presented in this study are available on request from the corresponding author. The data are not publicly available due to the privacy of participants and ethical concerns.

## Supplementary Materials

The following supporting information can be downloaded at: https://www.mdpi.com/article/10.3390/foods13010118/s1.
