# Supplementary material for: Raw Meat Consumption and Food Safety Challenges: A Survey of Knowledge, Attitudes, and Practices of Consumers in Lebanon
Source: Foods. 2023 Dec 29;13(1):118. doi: 10.3390/foods13010118 (PMC10779077; doi:10.3390/foods13010118)
Supplement: Supplementary file 1 [file foods-13-00118-s001.zip › foods-2748103-file s2.pdf]

1866 AUB
American University of Beirut
الجامعة الأميركية في بيروت

File S2. Questionnaire

Section 1 : Sociodemographic characteristics :

1. Gender
    a) Male
    b) Female
2. Age group
    a) 18-29
    b) 30-39
    c) 40 - 49
    d) 50-59
    e) >60
3. Area of Residency
    a) Beirut
    b) South
    c) North
    d) Mount Lebanon
    e) Bekaa
4. What is your highest Education level achieved?
    a) Primary school
    b) High school
    c) University degree (Bachelor)
    d) University degree (Masters)
    e) Technical degree
5. In the past 6 months have you or any one you know experienced one of the following: diarrhea, vomiting, fever, stomach cramps, nausea
    a) Yes
    b) No
6. What is the total monthly income of your household?
    a) <1000000 L.L
    b) 1000000 – 3000000 L.L
    c) 3000000 – 5000000 L.L
    d) >5000000 L.L
7. How do you rate your food safety knowledge?
    a) Excellent
    b) Good
    c) Weak
8. From where do you get your food safety information?
    a) Social media

b) Internet
c) Family or friends
d) TV
e) University
f) I don't have any source of information

Section 2: Knowledge about the risks associated with the consumption and safe handling of raw meat

1. Do you know that people can get seriously sick and die from eating contaminated food?
    a) Yes
    b) No
    c) I don't know
2. Do you know what food poisoning is?
    a) Yes
    b) No
    c) I don't know
3. Do you know which population is more susceptible to food poisoning?
    a) Children under 5 years' old
    b) Elderly
    c) Immuno-compromised individuals
    d) All
4. In your opinion can food get contaminated at:
    a) Market
    b) Home
    c) Both
5. The consumption of raw meat could pose serious economic losses to consumers
    a) Yes
    b) No
    c) I don't know
6. Form of cross contamination (transfer of microorganism) that could happen due to bad practices or bad storage conditions:
    a) From contaminated raw meat to other ready-to-eat food
    b) From a sick individual to raw meat
    c) Both
    d) I don't know
7. Can raw meat dish that looks and smells fresh make you sick?
    a) Yes
    b) No
    c) I don't know
8. Does freezing (<-18°C) prevent the growth of pathogens on raw meat?
    a) Yes, but it doesn't kill it
    b) No
    c) I don't know

9. The only way to ensure that you won't get food poisoned due to raw meat is
    a) Cook it at an appropriate temperature
    b) Freeze it
    c) I don't know
10. What is the optimal temperature for storing raw meat?
    a) <= 4°C
    b) >4°C
    c) I don't know
11. Regular washing of hands reduces the risk of contamination
    a) Yes
    b) No
    c) I don't know
12. Raw meat can be kept at room temperature for
    a) Less than 2 hours
    b) 2-4 hours
    c) More than 4 hours
    d) I don't know
13. During a long electricity cutoff, what should you do with your stored raw meat?
    a) Check its internal temperature and discard it if >4°C
    b) Keep it anyway
    c) I don't know
14. How long can you store raw meat in the fridge before consuming it?
    a) 3-5 days for most raw meat/ 1-2 days for ground meat and organ meat (ex: liver)
    b) 3-5 days for all
    c) 5-7 days for all
    d) I don't know
15. Do you know that some pathogens on raw meat can lead to kidney failures, sepsis, bloody diarrhea, and even death?
    a) Yes
    b) No
    c) I don't know
16. Parasites that could be found in raw meat dishes
    a) *Toxoplasma gondii*, that can lead to abortion in pregnant women
    b) *Trichinella spiralis*, that can lead in severe cases to various nervous disorders
    c) A and B
    d) Parasites are not found in meat
    e) I don't know
17. *Hepatitis A* virus that can cause liver inflammation can be found in raw meat dishes
    a) Yes
    b) No
    c) I don't know

18. *Helicobacter pylori* a bacterium that can cause peptic ulcer disease and gastritis can be transmitted through raw meat dishes
    a) Yes
    b) No
    c) I don't know
19. *Salmonella* bacteria that infect the intestinal tract can be found in raw meat dishes
    a) Yes
    b) No
    c) I don't know
20. *E. coli* O157: H7 can cause serious illness in humans by producing toxins that can severely damage the lining of your intestines and kidneys. Can you get this bacterium from eating raw meat dishes?
    a) Yes
    b) No
    c) I don't know
21. Campylobacteriosis (most common cause of bacterial diarrhea and can cause stunting in children) could be transmitted through raw meat dishes
    a) Yes
    b) No
    c) I don't know
22. Do you have any information on the consequences of antibiotic resistant bacteria that could be transmitted through food-raw meat?
    a) Yes
    b) No
    c) I don't know
23. Can contamination happen in unclean grounding machine?
    a) Yes
    b) No
    c) I don't know
24. Do you know that traditional ways of preparing raw meat dishes are more dangerous that modern ways?
    a) Yes
    b) No
25. Do you know that the longer the preparation time the more it is likely to contaminate the meat?
    a) Yes
    b) No
26. Is there any food safety law/ efforts in Lebanon that protects you as a consumer?
    a) Yes
    b) No
    c) There is a law but it is not implemented
    d) I don't know

Section 3: Attitude towards the risks associated with raw meat and safe meat handling

1. Using gloves is important in reducing the risk of food contamination

a) Agree
b) Disagree
c) I don't know

2. Preventing contamination is your responsibility
   a) Agree
   b) Disagree
3. Visual inspection is enough to determine if the food is safe
   a) Agree
   b) Disagree
4. Separate raw meat from ready-to-eat food to prevent cross contamination
   a) Agree
   b) Disagree
5. Your purchasing habits (places where you buy your meat, ex: meat stored in fresh air outside a butcher shop…) affects food safety
   a) Agree
   b) Disagree
6. People with high risk of contamination (children under 5 years old, elderly and immunocompromised individuals) should not consume raw meat
   a) Agree
   b) Disagree
7. Washing raw meat is an essential step
   a) Agree
   b) Disagree
8. Adding vegetables/spices to your raw meat at home could lead to cross-contamination
   a) Agree
   b) Disagree
9. Bacteria found on raw meat are not harmful
   a) Agree
   b) Disagree
10. Mincing the meat at home could have lower risk of contamination than buying already chopped and minced raw meat.
    a) Agree
    b) Disagree
11. Currently, how confident are you in the safety of raw meat in Lebanon?
    a) Very confident
    b) Confident
    c) Neutral
    d) Unconfident
    e) Very unconfident
12. Who do you think is responsible to for keeping the food safe?
    a) Government

b) Consumer
c) Market/ butcher shop
d) All

13. Do you prefer to buy raw meat for consumption imported or local?
    a) Imported
    b) Local
    c) Both
14. When you buy ground raw meat at the store or butcher shop, do you mind if they use grounding machine?
    a) Yes
    b) No
15. Why do you favor traditional preparation of raw meat?
    a) Taste
    b) Habit
    c) Texture
    d) I don't
16. Where do you prefer to buy your raw meat before processing it further at home?
    a) Market
    b) Butcher shops
17. Why do you prefer butcher shops?
    a) Trust
    b) Cleanliness
    c) Convenience
    d) Price
    e) Quality
    f) Other
18. Why do you prefer markets?
    a) Trust
    b) Cleanliness
    c) Convenience
    d) Price
    e) Quality
    f) Other
19. What is your favorite raw meat dish?
    a) Kibbeh
    b) Kaffta
    c) Liver
    d) All
20. If you are aware of the risks associated with the consumption of raw meat would you refrain from eating it?
    a) Yes
    b) No

c) I don’t know

Section 4: Practices that could increase the risk of foodborne infection

1. How often do you eat raw meat dishes per week?
    a) Not every week
    b) Once per week
    c) More than once per week
2. During your supermarket shopping, when do you buy raw meat
    a) The first thing I buy is raw meat
    b) The last thing I buy is raw meat
    c) I don’t follow any order
3. Do you use a thermometer while cooking the meat?
    a) Yes
    b) No
4. Do you prepare and handle raw meat while you are sick?
    a) Yes
    b) No
5. Do you wear gloves while preparing your food?
    a) Yes
    b) No
6. Do you wash your hands before and after handling raw meat?
    a) Yes
    b) No
7. After preparing raw meat, how do you clean your kitchen counters?
    a) Using water
    b) Water, then detergent
    c) Water, then sanitizer
    d) Detergent, then water, then sanitizer
8. Do you check the temperature of your refrigerator often during the day?
    a) Yes
    b) No
9. Where do you store your raw meat in the refrigerator?
    a) Upper shelf
    b) Middle shelf
    c) Lower shelf
10. After the crisis, did you aim for meat with lower prices?
    a) Yes
    b) No
11. How do you defrost frozen meat?
    a) In the microwave
    b) Leave it on the kitchen counter until defrosted

    c) Under running water
    d) Leave it in the refrigerator one night before.

12. Would you offer raw meat to children and the elderly in your household?
    a) Yes
    b) No
13. Do you prepare your own raw meat dishes and/ or eat them at a restaurant?
    a) Yes
    b) No
14. How likely are you to eat raw meat dishes outside your home/ restaurant?
    a) Very likely
    b) Likely
    c) Neutral
    d) Unlikely
    e) Very unlikely
15. If you prepare raw meat dishes (kibbeh) at home, do you use:
    a) Traditional way using hammer and stone
    b) Mechanical modern machines
    c) I don't prepare at home
16. If you use traditional wats, does your mortar (mdakka) and pestle (tabblieh/ journ) have any cracks?
    a) Yes
    b) No
17. How long does it normally take you to prepare a raw meat dish (kibbeh)?
    a) 30 minutes or less
    b) 30 minutes to 1 hour
    c) 1-2h
    d) More than 2h
18. Do you look for food safety certificates in the market or shop where you buy your meat?
    a) Yes
    b) No
